# Evidence-Informed Practice: Diagnostic Questions in Urinary Tract Infections in the Elderly

**DOI:** 10.5811/westjem.2019.5.42096

**Published:** 2019-06-11

**Authors:** Richard Pescatore, Joshua D. Niforatos, Salim Rezaie, Anand Swaminathan

**Affiliations:** * Crozer-Keystone Health System, Department of Emergency Medicine, Upland, Pennsylvania; † Cleveland Clinic Lerner College of Medicine of Case Western Reserve University, Department of Emergency Medicine Cleveland, Ohio; ‡ Greater San Antonio Emergency Physicians, Department of Emergency Medicine, San Antonio, Texas; § St. Joseph’s Regional Medical Center, Department of Emergency Medicine, Paterson, New Jersey

## Abstract

**Introduction:**

Routine interventions in the practice of medicine often lack definitive evidence or are based on evidence that is either not high quality or of only modest-to-marginal effect sizes. An abnormal urinalysis in an elderly patient presenting to the emergency department (ED) with non-specific symptoms represents one condition that requires an evidence-informed approach to diagnosis and management of either asymptomatic bacteriuria or urinary tract infection (UTI). The emergency provider often will not have access to urine cultures, and the risks associated with antibiotic use in the elderly are not without potentially significant side effects.

**Methods:**

We performed a historical and clinical review of the growing body of literature suggesting measurable differences in the systemic immune response manifest among patients with asymptomatic pyuria and UTI, including increases in the pro-inflammatory cytokine interleukin-6 and the acute phase reactant procalcitonin.

**Results:**

Serum procalcitonin, a peptide that undergoes proteolysis into calcitonin, has been demonstrated to quickly and reliably rise in patients with severe bacterial infections, and may serve as a potentially sensitive and specific marker for identification of bacterial illness.

**Conclusion:**

In the absence of validated risk scores for diagnosing UTI in elderly patients presenting to the ED, there may be a role for the use of procalcitonin in this patient population.

## INTRODUCTION

### Evidence-Based to Evidence-Informed Practice: The Clinical Reality

The evolution from eminence-based to evidence-based care has come to define bedside emergency medicine (EM), with rigorous skepticism and scholarly consideration accelerated by the power of global connectivity.^1^ Where anecdote and opinion once drove therapy, clinicians now approach clinical conundrums with deliberate reflection, expecting – and at times demanding – ever-higher proof of perfection prior to implementing or incorporating therapies, tests, or approaches into their own practice. Such cogitation ensures excellence and safety and avoids pitfalls of over-adoption or confounding. Sackett originally defined this approach as evidence-based medicine, or the “use of current best evidence in making decisions about the care of individual patients...integrating individual clinical expertise” and honoring patients’ values and preferences.^2^ Unfortunately, many of our daily decisions are made in a space devoid of definitive data.^3^–^8^

We thus are required to transition from the ideals of evidence-*based* medicine into the real and pragmatic world of evidence*-informed* medicine*.* It is at this precipice of real-world practice—where often studies and statistics do not exist, are not of high quality, or are of modest-to-marginal effect sizes—that we change, where we push forward the boundaries of care, and develop not only experience but the very questions that will define the next advances in EM. With this in mind, we sought to explore the clinical realities of the assessment and treatment of abnormal urinalyses in the elderly patient presenting with non-specific symptoms to the emergency department (ED).

## METHODS

We performed a historical and clinical review of the current literature on urinary tract infection (UTI) focusing on identifying and summarizing key trends relevant to the diagnosis of abnormal urinalyses in elderly patients with non-specific symptoms with a specific emphasis on the biomarker procalcitonin.

## RESULTS

### Asymptomatic Bacteriuria: Historical Perspective

Asymptomatic bacteriuria was first identified in the mid-1950s, when a series of autopsy reports identified chronic pyelonephritis as a common cause of renal failure, despite the fact that none of the deceased individuals had been diagnosed with a urinary tract infection while alive.^9^ Such findings led researchers to declare, “there is now clear evidence that bacteriuria is one of the commonest human infections, that it may be chronic and persistent, that it may influence structure and function outside of the urinary tract, and that it plays an important role in disease from the cradle to the grave—from prematurity to hypertension and renal failure.”^10^ Since these observations, decades of antibiotics have been administered with frequency through the mouths, veins, and bladders of asymptomatic patients, all while several studies conclusively demonstrated that treatment of asymptomatic bacteriuria not only lacks benefit, but likely increases the short-term risk of pyelonephritis.^11^

### Asymptomatic Bacteriuria: Clinical Pearls in the Emergency Department

Asymptomatic bacteriuria is a microbiologic diagnosis defined as >10^5^ colony-forming units per milliliter (cfu/mL) bacteria identified in two consecutive voided urine specimens (only one specimen needed for males).^12^ It is incredibly common, affecting greater than 20% of healthy, elderly, community-dwelling women, and reaching a prevalence as high as 50% in institutionalized elderly females, and 100% in patients with indwelling catheters.^13^ In the ED, without access to culture information, emergency providers are thus not confronted with asymptomatic bacteriuria, but rather asymptomatic *pyuria*, an even more prevalent and low-yield urinalysis usurper.

The clinical conundrum of the abnormal urinalysis in an elderly patient presenting with non-specific symptoms is a common occurrence.^14^ While it is well-known that UTIs can cause malaise and mental status changes in elderly populations,^15^ consistent clinical data suggest that the overwhelming percentage of “positive” urinalyses (UA) represent asymptomatic bacteriuria, and are neither truly indicative of UTI nor related to the patient’s non-specific complaints.^16^ Additionally, fixating on the “positive” UA can often cause clinicians to reach premature diagnostic closure and stop further investigations. Unfortunately, determining which UAs represent constitution-influencing infection and which are simple asymptomatic presentations is a difficult task in the ED, particularly when the patient lacks the cognitive or physical ability to relay typical symptoms such as dysuria, urinary frequency, or hesitancy.

Knowing that antibiotic treatment is of no value in non-pregnant patients with simple asymptomatic bacteriuria,^11^,^12^ we strive to limit such unnecessary antimicrobials, which also bring risk of allergic reaction, increased antibiotic resistance, and iatrogenic injury ranging from *Clostridium difficile* colitis to renal injury.

Despite compelling data that treatment of asymptomatic bacteriuria lacks benefit, and growing evidence that non-specific symptoms in the elderly are generally *not* caused by UTIs,^16^ these patients are overwhelmingly exposed to broad spectrum agents initiated in the ED or upon admission to the hospital.^17^,^18^ It can be difficult for the emergency provider to avoid incorporating into the diagnostic framework the need for antibiotic stewardship with the real concerns of disease progression or missed infection treatment metrics. Even when restraint wins out in the ED, diagnostic equipoise often drives inpatient teams to start antibiotics. Strikingly, well-done surveys demonstrate that nearly half of physician respondents prescribe antibiotics for asymptomatic bacteriuria despite *knowing* a UA demonstrates asymptomatic bacteriuria and not true infection.^19^ When clinicians are unable to elicit information on lower urinary tract symptoms from elderly patients with dementia, advanced cerebrovascular disease, or other impairments resulting in communication barriers, they are likely to initiate antimicrobial therapy in a well-intentioned effort to prevent progression and worsening infectious outcomes with anecdote of improvement perpetuating such practice.^20^

### Systemic Immune Response

Recognizing the difficulty in distinguishing true infection from asymptomatic bacteriuria, multiple authors have attempted to investigate differences in systemic immune response among these cohorts. In 2011, a group of researchers compared the levels of various inflammatory markers in urine samples before and after inoculation with *Escherichia coli*, noting a substantial increase in both white blood cells and polymorphonuclear neutrophils, a not uncommon phenomenon in lab results seen in the ED.^21^ Interestingly, however, levels of interleukin-6 (IL-6), a pro-inflammatory cytokine that is not only an acute phase reactant but also one of the major regulators of acute phase protein synthesis,^22^,^23^ remained unchanged in this study.^21^ The authors suggested that these findings support the hypothesis that a less-robust host immune response occurs among patients with asymptomatic bacteriuria compared to those with symptomatic infection.

A subsequent study exploring the role of IL-6 and heparin binding-protein (HBP), a protein released from activated neutrophils during infection^24^ and previously shown to be associated with UTIs,^25^ a 2016 investigation enrolled asymptomatic, elderly, nursing home residents and matched them with patients living in the community or at nursing homes with symptomatic UTI.^26^ In this study, urinary IL-6, but not HBP, reliably distinguished between patients with cystitis or asymptomatic bacteriuria, adding further to the growing observational literature that there is a fundamental *and measurable* difference in the body’s immune reaction in cases of true infection compared to colonization.^26^

### Procalcitonin and Urinary Tract Infection

Perhaps one of the most hotly debated acute phase reactants, procalcitonin, is the latest diagnostic darling in inpatient circles. Procalcitonin (the biologic precursor to calcitonin) reaches measurable serum concentrations quickly and reliably in the setting of bacterial infection.^27^–^29^ Dozens of trials have reliably demonstrated procalcitonin’s strong performance in decision support for antibiotic initiation or cessation; however, in the most salient ED investigation—the Procalcitonin Consensus Trial (ProACT) —procalcitonin failed miserably in limiting unnecessary antibiotic use.^30^ Notably, however, such failure seemed more a function of clinician fears of untreated infection rather than a shortcoming of the test itself, an idiosyncrasy noted many times over in a myriad of editorials following publication of ProACT. The recently published HI-TEMP trial, however, applied PCT across a heterogeneous population and found no benefit in decreasing antibiotic use or significantly decreasing patient-oriented endpoints.^31^ As many respondents to ProACT argued, though – and the trial authors seemed to agree with – the combination of this reassuring objective test with a concerted effort to limit unnecessary antibiotic use, or an antibiotic stewardship program, could be more effective in harnessing the diagnostic value of procalcitonin.^32^–^34^

## DISCUSSION

We suggest that a considered and nuanced synthesis of this inflammatory marker in a well-defined clinical context is supported by a vibrant body of literature suggesting a role for procalcitonin in the diagnosis of true UTI. In one bench study, procalcitonin reliably increased with worsening urinary tract disease.^35^ Additionally, one comprehensive review found procalcitonin to be a “key marker” in children with UTI.^36^ When operationalized, serum procalcitonin was a good predictor of disease severity in a meta-analysis of prospective pediatric clinical trials of UTIs.^37^ More recently, a randomized controlled trial (RCT) out of Switzerland randomized patients presenting to the ED with a UTI to a procalcitonin-pyuria-based (PCT-pyuria) algorithm or current guidelines (control group) for initiation and duration of antibiotic therapy.^38^ In the intention-to-treat analysis, cumulative 90-day exposure to antibiotics was shorter in the PCT-pyuria group compared to the control, although with no changes in mortality and reinfections. In a 2015 *Cochrane* review assessing procalcitonin for diagnosing acute pyelonephritis in pediatric patients,^39^ there were limited studies at the time of the review and marked heterogeneity of the included studies to recommend it for daily practice, although procalcitonin performed significantly better for ruling-in pyelonephritis compared to erythrocyte sedimentation rate or C-reactive protein.

It is important to note that procalcitonin can be elevated in any acute infectious circumstance and is not specific to a UTI. Therefore, an elevated procalcitonin level should not be equated to UTI and does not clinch the diagnosis of UTI. Pyuria in the setting of an elevated procalcitonin level may still represent asymptomatic bacteriuria with an alternate infectious process (i.e., meningitis, bacteremia*,* etc.). Clinicians should be cautious to avoid premature diagnostic closure in this circumstance. Furthermore, it should be noted that PCT levels may not rise with localized infections (septic arthritis, localized abscess, etc.), steroid use, and atypical bacteria,^40^,^41^ and decisions regarding antimicrobial therapy should not be based solely on procalcitonin serum concentrations.

### Procalcitonin for Urinary Tract Infection in the Emergency Department

The management morass of abnormal urinalyses in the elderly patient with nonspecific symptoms may represent an excellent opportunity for utilization of procalcitonin testing using an evidence-informed approach, ie, efforts undertaken in the absence of strict guiding data but supported and shepherded by complementary knowledge. Given the ambiguity of asymptomatic bacteriuria, a de-facto antibiotic stewardship effort is already underway in every hospital across the country, as well-meaning physicians strive to separate true infection from asymptomatic distractors. Where clinical complacency exists—an afebrile, non-toxic-appearing patient in whom the desire to spare unnecessary antibiotic use conflicts with the compulsion to not allow an indolent infection run rampant—a procalcitonin-augmented strategy might satisfy both imperatives.

This recommended strategy is not novel. As recently reported in the *American Journal of Emergency Medicine*, one retrospective analysis of UTIs found a negative predictive value of 91% for a low procalcitonin further bolstering the argument in favor of its use as an adjunct in the non-initiation of empiric antibiotics.^42^ Even more compelling, a RCT of nearly 200 ED patients found that a procalcitonin-based algorithm reduced antibiotic exposure by 30% without negative effects on clinical outcomes.^38^ The introduction of procalcitonin in these departments served as a reliable and objective diagnostic marker and limited costly, harmful, and unnecessary exposure to antibiotics.

## LIMITATIONS

The use of procalcitonin in the work-up of UTI in elderly patients with an abnormal urinalysis presenting with non-specific symptoms requires further investigation ideally through a multicenter, RCT. Furthermore, this historical and clinical review was not systematic in its goal to describe the entirety of the use of procalcitonin; however, the purpose of this paper was to provide a succinct, narrative update of the latest research related to the use of biomarkers for diagnosing UTI for this patient population in the ED setting.

## CONCLUSION

Where definitive studies are lacking, nuance and rational integration of the literature is not only an option, but an imperative. The application of evidence-based medicine is at its easiest after large RCTs and rigorous analyses are popularized and widely disseminated. A true test of bedside Bayesianism, however, comes when the clinician is presented with clinical conundrums not yet thoroughly vetted and extensively analyzed. When no clear answer exists in the literature, we are forced to faithfully apply the best available knowledge to answer critical questions in real time in an ongoing attempt to correct shortcomings and pursue better care. No current rigorous trial has yet examined procalcitonin’s performance in this narrow and nuanced framework, but the collated and considered information available suggests that adding this simple test likely provides a much-needed diagnostic beacon and can safely lead to better care in real-world applications.
